# Relation of Chlorophyll Fluorescence Sensitive Reflectance Ratios to Carbon Flux Measurements of Montanne Grassland and Norway Spruce Forest Ecosystems in the Temperate Zone

**DOI:** 10.1100/2012/705872

**Published:** 2012-06-04

**Authors:** Alexander Ač, Zbyněk Malenovský, Otmar Urban, Jan Hanuš, Martina Zitová, Martin Navrátil, Martina Vráblová, Julie Olejníčková, Vladimír Špunda, Michal Marek

**Affiliations:** ^1^Global Change Research Centre AS CR, Bělidla 4a, 603 00 Brno, Czech Republic; ^2^Remote Sensing Laboratories, Department of Geography, University of Zürich, Winterthurerstrasse 190, 8057 Zürich, Switzerland; ^3^Department of Surveying and Spacial Sciences, School of Geography and Environmental Studies, University of Tasmania, Private Bag 76, Hobart TAS 7001, Australia; ^4^Department of Physics, Faculty of Science, University of Ostrava, 30. dubna 22, 701 03 Ostrava, Czech Republic; ^5^Department of Plant Physiology, Faculty of Science, University of South Bohemia, Branišovská 31, 370 05 České Budějovice, Czech Republic

## Abstract

We explored ability of reflectance vegetation indexes (VIs) related to chlorophyll fluorescence emission (*R*_686_/*R*_630_, *R*_740_/*R*_800_) and de-epoxidation state of xanthophyll cycle pigments (PRI, calculated as (*R*_531_ − *R*_570_)/(*R*_531_ − *R*_570_)) to track changes in the CO_2_ assimilation rate and Light Use Efficiency (LUE) in montane grassland and Norway spruce forest ecosystems, both at leaf and also canopy level. VIs were measured at two research plots using a ground-based high spatial/spectral resolution imaging spectroscopy technique. No significant relationship between VIs and leaf light-saturated CO_2_ assimilation (*A*_MAX_) was detected in instantaneous measurements of grassland under steady-state irradiance conditions. Once the temporal dimension and daily irradiance variation were included into the experimental setup, statistically significant changes in VIs related to tested physiological parameters were revealed. ΔPRI and Δ(*R*_686_/*R*_630_) of grassland plant leaves under dark-to-full sunlight transition in the scale of minutes were significantly related to *A*_MAX_ (*R* ^2^ = 0.51). In the daily course, the variation of VIs measured in one-hour intervals correlated well with the variation of Gross Primary Production (GPP), Net Ecosystem Exchange (NEE), and LUE estimated via the eddy-covariance flux tower. Statistical results were weaker in the case of the grassland ecosystem, with the strongest statistical relation of the index *R*_686_/*R*_630_ with NEE and GPP.

## 1. Introduction

The consequences of the ongoing global climate change [1] might turn terrestrial ecosystems over large areas from carbon sink to carbon source [2–4]; therefore, an accurate assessment of the global carbon balance is of increasing importance. Remote sensing (RS) provides the only global and cost effective tool to monitor spatiotemporal properties of the CO_2_ assimilation in various plant ecosystems [5–9]. One of the traditional approaches to quantify RS information is the transformation of reflectance spectra into vegetation indexes (VIs). VIs are mathematical transformations of reflectance at specifically selected spectral bands that maximize sensitivity to target biophysical variables and minimize confounding environmental factors (e.g., [10]). Since the start of the satellite era-several VIs have been developed for remote quantification of leaf chlorophyll content (Chl_(*a*+*b*)_) [11], leaf area index [12], and/or other biophysical variables [13] that are important for the assessment of the health status and functioning of terrestrial ecosystems. However, most of these derived variables and VIs are rather insensitive to daily dynamical changes in ecosystem carbon fluxes (however, see [14]).

Recent advances in RS provided the possibility to estimate physiological parameters related to the dynamic processes of CO_2_ fixation by photosynthesis [15–17], namely, to (i) xanthophyll cycle pigments and (ii) chlorophyll fluorescence (Chl-F). Such approach of direct estimation of the light use efficiency (LUE) has a potential to contribute to a significant reduction of existing large uncertainties in the estimation of the carbon balance [16].

The xanthophyll cycle pigments protect the photosynthetic apparatus from overexcitation [18]. Under high light conditions, plants activate a photoprotective pathway via deepoxidative reactions of xanthophyll pigments, resulting in zeaxanthin as the final product (e.g., [19]). This process enables the dissipation of excessive energy in the form of heat [20] and is associated, along with other processes, with the reduction in photochemical efficiency of Photosystem II (PSII) [18]. These rapid and light intensity-dependent changes were firstly detected remotely as changes in absorbance around 515 nm [21] and later as changes in reflectance around 530 nm [22]. Subsequently, Gamon et al. [23] proposed Photochemical Reflectance Index (also called Plant Physiological Reflectance Index, PRI, calculated as (*R_531 _*− *R_570_*)/(*R_531 _*+ *R_570_*)), where *R*_*XXX*_ is a reflectance intensity at subscripted wavelength. This index has been related to changes in the zeaxanthin pigment content [23–25], LUE (defined as the amount of assimilated carbon per unit of incoming light) measured at leaf [25] and later also at canopy [26–30] and even at satellite levels [7, 31, 32]. Several studies linked PRI measured by eddy-covariance flux towers directly to carbon sink capacity in grassland [33] and forest ecosystems [34]. PRI index has been also shown to correlate with the foliar pigment content of Chl_(*a*+*b*)_, carotenoids (Car_*x*+*c*_), Car/Chl ratio and Car/Chl ratio [35, 36] and various Chl-F parameters, especially under the drought stress conditions [37–40].

The Chl-F emission is directly linked to the first steps of photosynthesis, to photochemistry, and it is widely used in photosynthesis research. Chl-F is produced in plant photosynthetic tissues after the absorption of light energy as one of the two deexcitation pathways competing with photochemistry and heat loss [41–43]. The coupling between the photochemistry and Chl-F is the strongest in PSII. Thus, the competition between Chl-F and photosynthesis makes the Chl-F an ideal noninvasive reporter of the photosynthetic activity in plant tissue. The Chl-F emission reaches its maximum intensity at 690 and 740 nm [44]. Also changes in the zeaxanthin concentration are related to Chl-F, particularly to Nonphotochemical Quenching (NPQ) [45].

Chl-F expresses dynamic changes when a previously dark adapted healthy plant is exposed to actinic light [33]. After the illumination Chl-F increases to a high peak value, it then decreases to a steady state level (F*_S_*) [46]. Under saturating light, the peak Chl-F equals to the maximum level (F*_M_*). The relative Chl-F decrease (R_Fd_) from the dark-to-light adapted state, quantified as R_Fd_ = (F*_M_* − F*_S_*)/F*_S_*, was shown to be linearly correlated with the light-saturated photosynthetic rate [47].

However, the only sun-induced Chl-F signal that can be tracked via passive RS sensors is “*steady-state*” chlorophyll fluorescence (Chl-F*_S_*) [48–50]. Results of several studies suggested that solar-induced Chl-F*_S_* [51–55] is linked to CO_2_ assimilation. Consequently, several VIs, particularly, the reflectance ratios *R_686_*/*R_630_*, *R_690_*/*R_630_*, and *R_740_*/*R_800_* have been proposed to quantify Chl-F*_S_* [37, 50], where the first wavelength corresponds to the maximum intensity of Chl-F*_S_* emission, whereas the second wavelength is unaffected by Chl-F*_S_* thus serving as a normalization factor. The difference ratio *R_760.59_* − *R_759.5_* utilizes the Chl-F infilling effect [56].

The Frauenhofer Line Discriminator principle [57] is another method used in RS for Chl-F*_S_* signal extraction from reflectance measurements [34]. Nevertheless, most of the studies using the retrieval of Chl-F*_S_* from hyperspectral reflectance were carried out either in laboratory conditions or with nonimaging sensors. Only a limited number of studies attempted to map the spatial distribution of carbon fluxes which were implemented using ground-based [58, 59] airborne [60, 61] or spaceborne [7, 31] spectroscopy sensors.

In this study, we explored the ability of reflectance VIs related to the F*_S_* signal, such as *R_686_*/*R_630_*, *R_740_*/*R_800_* and PRI, to estimate the CO_2_ assimilation at canopy level in grassland and forest ecosystems. First, we evaluated whether the heterogeneity of VIs of a measured grassland plot can reveal a heterogeneity in light-saturated CO_2_ assimilation rates (*A*_MAX_) under steady state conditions. Second, we followed VI changes under a change of irradiance intensity in the scale of minutes and hours and searched for correlations between VI variations and variations of physiological variables, particularly LUE, gLUE (gross light use efficiency; GPP/APAR), net ecosystem exchange (NEE) and gross primary production (GPP), measured using the eddy-covariance systems at the scale of grassland and forest ecosystems. The aim of this study was to explore potentials and limitations of scaling the carbon cycle-related physiological processes, observable via RS methods, from leaf to canopy level. This way the feasibility of larger scale RS observation of the vegetation processes at ecosystem level can be advanced. To our knowledge, this is the first study using the imaging spectroscopy to investigate plant canopy properties in relation to eddy-covariance data in the diurnal course [17]. 

## 2. Material and Methods

### 2.1. Study Site: Montane Grassland and Forest Ecosystems

The montane grassland and forest research plots are located at the experimental study site Bílý Kříž (Moravian-Silesian Beskydy Mts., the Czech Republic, 18.54°E, 49.49°N, 855 m and 908 m a.s.l., resp.), which is part of the CARBOEUROPE flux tower network (http://www.carboeurope.org/). This site is characterized by cool (annual mean temperature of 5.5°C) and humid (annual mean relative air humidity of about 80%) climate with high annual precipitation (ca. 1000–1400 mm). The geological bedrock is formed by Mesozoic Godula sandstone (flysch type) with ferric podzols.

The most abundant species occurring at the natural grassland plot (association *Nardo-Callunetea*, class *Nardo-Agrostion tenuis*) that were investigated are *Festuca rubra agg. *(L.),* Hieracium sp., Plantago sp., Nardus stricta *(L.), and* Jacea pseudophrygia* (C. A. Meyer). Additional details on the experimental grassland and physiological properties of the dominant plant species are presented in Urban et al. [62].

The investigated forest stand was planted in 1981 with 4-year old seedlings of *Picea abies *(L.) *Karst.* (99%) and *Abies alba* (Mill). (1%) on a slope of 11–16° with south-southwest exposition over an area of 0.062 km^2^ [63]. At the time of the experimental measurements (2005), the stand density was approximately 2 600 trees/ha (hemisurface leaf area index of about 11 m^2^ m^−2^), with mean (± standard deviation) tree height of 8.5 m (±1 mm) and stem diameter at 1.3 m of 10.1 m ± (1 mm).

### 2.2. Imaging Spectroscopy Measurements and Data Processing

**Figure 1:**
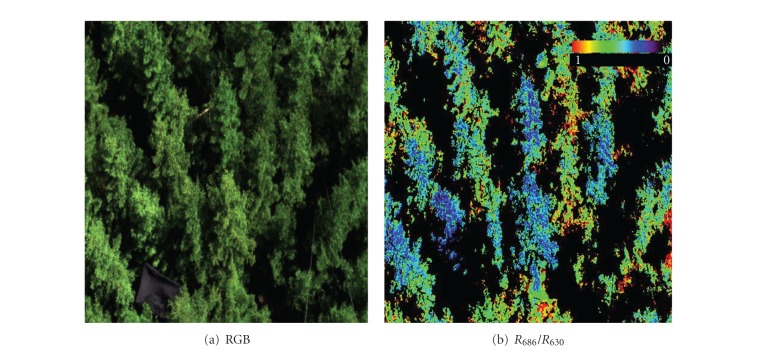
The image shows a true color red, green, and blue (RGB) composition of a Spruce forest measured in the Beskids Mountains acquired on 2nd September of 2005 at 10:00 A.M. (a) The same image was converted into the reflectance ratio *R_686_*/*R_630_* where the pixels in the shadow and with nongreen vegetation are excluded from the image and further analysis (b).

The nadir viewing canopy reflectance was obtained under clear sky sunny conditions with a visible near-infrared hyperspectral Airborne Imaging Spectrometer for Applications (AISA Eagle, Specim, Oulu, FI). In total 260 spectral bands between 400–940 nm were collected with a full width at half maximum (FWHM) of 2.2 nm, and with a ground pixel resolution of 1 cm. The AISA sensor was mounted to the tower at a height of 20 meters (i.e., approximately 10 m above the top of the canopy). The acquired area of about 50 m^2^ was located within a footprint of an eddy-covariance tower system. In the case of grassland, the AISA sensor was attached to a ladder at the height of 4 meters above the top of the canopy, sensing in a total area of about 4 m^2^ (located completely within a footprint of an eddy-covariance system), with a ground pixel resolution of about 0.3 cm.  Figure 1. shows the representative part of the Norway spruce forest, which is homogeneous even-aged monoculture, thus the assumption here is that the smaller footprint of reflectance measurements is possible to be compared with the larger footprint of eddy-covariance data. Subsequent airborne measurement covering the whole footprint area has confirmed this assumption for both forest as well as grassland ecosystem (data not shown).

The acquired AISA images were transformed into radiance values using the laboratory-derived calibration files, and converted into at-sensor reflectance images (Figure 1(a)) by means of the empirical line method, using five near-Lambertian calibration panels of known flat reflectance response. An automatic supervised Maximum Likelihood classification [64] was used to distinguish and to mask shaded pixels. To ensure that only photosynthetically active pixels were used in the subsequent statistical analysis, an appropriate threshold of the green normalized difference VI (green NDVI = (*R_554_* − *R_677_*)/(*R_554_* + *R_677_*)) was applied to separate the green sunlit grass leaves from dry litter. Finally, three narrow-band VIs (PRI, *R_686_*/*R_630_*, *R_740_*/*R_800_*) related to photosynthetic processes were derived per pixel from the AISA Eagle reflectance scans (Figure 1(b), Table 1). Selection of these VIs is based on our previous work with AISA Eagle, where detailed diurnal hyperspectral measurements were coupled with Chl-F measurements (see [48] for full description). No other optical VIs were analyzed in this study.

### 2.3. Leaf Gas Exchange Measurements of Grassland Species

The *in situ* CO_2_ assimilation rate of intact leaves of *Festuca rubra agg.*,* Hieracium sp., Jacea pseudophrygia*, *Plantago lanceolata*, *Tarraxacum spp*., and *Veronica chamaedrys* (L.) was measured at saturating light intensities (Photosynthetic Photons Flux Density (PPFD) *≈* 1300 ± 10 *μ*mol (photons) m^2^ s^−1^, [63]) with the open infrared gasometrical analyzer Li-6400 (Li-Cor, USA). All environmental conditions inside the assimilation chamber remained unchanged and referred to the field conditions: leaf temperature between 34–37°C, relative humidity between 35–45%. All leaves, still being attached to the plant, were measured in their natural environment and marked. This way, they were later identified on the AISA images, and all corresponding green pixels were averaged in order to minimize the angular anisotropy of the vegetation bidirectional reflectance distribution function (BRDF).

### 2.4. Canopy Eddy-Covariance Measurements

Fluxes of CO_2_ and H_2_O, as well as latent and sensible heat exchange between the grass stand and atmosphere, were measured using closed-path eddy-covariance systems Edisol (University of Edinburgh), containing the infrared gas analyzer (Li-6262, Li-Cor, Lincoln, USA), sonic anemometer Gill *R* ^2^ (Gill Instruments, UK), and Edisol software package. In the case of the forest research stand, the InSituFlux (InSituFlux, Sweden), consisting of the infrared gas analyzer (LI-7000 Li-Cor, Lincoln, USA), anemometer (Gill R3, Gill Instruments Ltd., Lymington, UK) and EcoFlux software, was used. The eddy-covariance systems provide time series of half an hour integral measurements of CO_2_ and H_2_O fluxes. The footprint of the system covers a circular area with a radius of ca 500 m [65]. The postprocessing of eddy-covariance data was carried out based on the methodology paper Aubinet et al. [66], accommodating several modifications according to the most recent CarboEurope and FLUXNET protocols. The Quality Control (QC) Software performed the spike removal and quality check of the raw signals. After gap filling the GPP and NEE, values were modeled according to Urban et al. [63]. LUE and gLUE were calculated as follows: LUE = NEE/PPFD and gLUE = GPP/PPFD, where PPFD stands for Photosynthetic Photons Flux Density. For LUE and gLUE calculations we used PPFD values instead of commonly used absorbed PPFD, since the amount of transmitted light through the young dense forest stand is negligible (<2%; data not shown).

### 2.5. Laboratory Analysis of Foliar Pigments

Plant leaves of grassland (*n* = 24) and current one-year and two-year old needles of Norway spruce trees (*Picea abies *(L.)* Karst.*, 100 mg, *n* = 6) were sampled after the spectral measurements during the morning, midday, and afternoon (i.e., at 9:00, 12:00 and 16:00 of local time). Foliar pigments of samples, frozen into laboratory in liquid nitrogen for transportation, were extracted in 80% acetone with a small amount of MgCO_3_. After centrifugation at 480 g for 3 min. the supernatant was used for spectrophotometric (UV/VIS 550, Unicam, Leeds, UK) estimation of chlorophyll *a* and *b per area*, according to Lichtenthaler [67], and also for High-Performance Liquid Chromatography (HPLC) quantification (TSP Analytical, USA) of individual carotenoids, as described in Štroch et al. [68]. The deepoxidation state of the xanthophyll cycle pigments (DEPS) [69] was calculated using conversion factors published by Farber [70] as DEPS = (Z + A)/(V + A + Z) [71], where Z is zeaxanthin, A is antheraxanthin, and V is the violaxanthin content.

### 2.6. Setup of Field Experiments

The variation of VIs was measured under three irradiance regimes: (i) instantaneous steady-state measurements under full sun irradiation, (ii) variation of VIs within dark-to-light transition in scale of minutes, and (iii) daily variation of VIs measured in one-hour intervals.

Within the frame of the “*steady-state*” experiment (i) we mapped VIs of selected experimental grassland plots (an area of about 4 m^2^) at midday under full sunlight. 24 leaves of 6 plant species were labeled (each leaf was located on a separate plant), and their maximal CO_2_ assimilation rate (*A*_MAX_) was measured under saturating midday irradiance (PPFD *≈* 1300 ± 10 *μ*mol (photons) m^−2^ s^−1^). These leaves were identified on the AISA image, captured simultaneously with CO_2_ assimilation rate measurements. The computed VIs of 10–20 sunlit pixels of each leaf were averaged and correlated to the *A*_MAX_ measurements. The experiment was carried out on a clear-sky day on 18th of July 2007 at 13:00 local time.

In the “*shade removal*” experiment (ii), run in a time scale of minutes, we tested whether the VIs could estimate the CO_2_ assimilation rate of labeled grass leaves exposed to saturating sun irradiation (*A*_MAX_) not in the static steady state, but in a dynamic irradiation environment inducing the plant photoprotective reactions and consequent onset of photosynthesis. The experimental plot was divided into two parts (twice 0.5 × 4 m). The first part, containing 13 labeled sample leaves, was covered for 30 min using a black nontransparent blanket in order to dark-adapt the grass canopy prior to the imaging spectroscopy measurement. Our method of darkening enabled a lateral air convection, which prevented the physiological changes being induced by higher than ambient air temperature. The second control plot was exposed to a natural irradiation regime. Two AISA Eagle images of both plots were obtained at the 5th and 90th seconds after blanket removal. Subsequently, we computed the dynamic change of the VIs (ΔVIs) during the dark-to-light transition per leaf sample (average of 10–20 pixels) as subtraction VI_DARK_ − VI_LIGHT_, where VI_DARK_ and VI_LIGHT_ are values obtained from the scan taken ~5 and ~90 seconds after removal of the blanket, respectively. These ΔVIs were statistically related to *A*_MAX_ measurements of the sampled leaves.

 Finally, the “*daily course*” experiment (iii), conducted at a time scale of hours, was to correlate the daily variations of the VIs' mean values of both grassland and forest ecosystems with a daily variation of physiological variables measured in half-hour intervals via eddy-covariance flux systems. Grassland hyperspectral image data were obtained on the September 2, 2005 in one-hour intervals from 9:30 to 16:00 local time. The forest measurements were carried out at one-hour intervals from 13:30 to 16:30 on August 30, 2005 and on August 31, 2005 from 9:30 to 16:30 local time. The daily variations of the selected VIs measured as well as the carbon fluxes were jointly investigated for grassland and forest ecosystems.

### 2.7. Statistical Analysis

The best-fitting models were computed to evaluate the relationship between the VIs and the physiological variables of carbon assimilation. The amount of variability of a dependent variable explained by the independent variable within the established regression model was expressed by the coefficient of determination (*R* ^2^). The significance of the relationships between the variables was tested using the analysis of variance (ANOVA) at two significance levels: *P* < 0.1 and *P* < 0.05, respectively. All statistical tests were performed with Statistica 7.0 software (StatSoft Inc., USA).

## 3. Results

### 3.1. “Steady-State” Experiment

**Figure 2:**
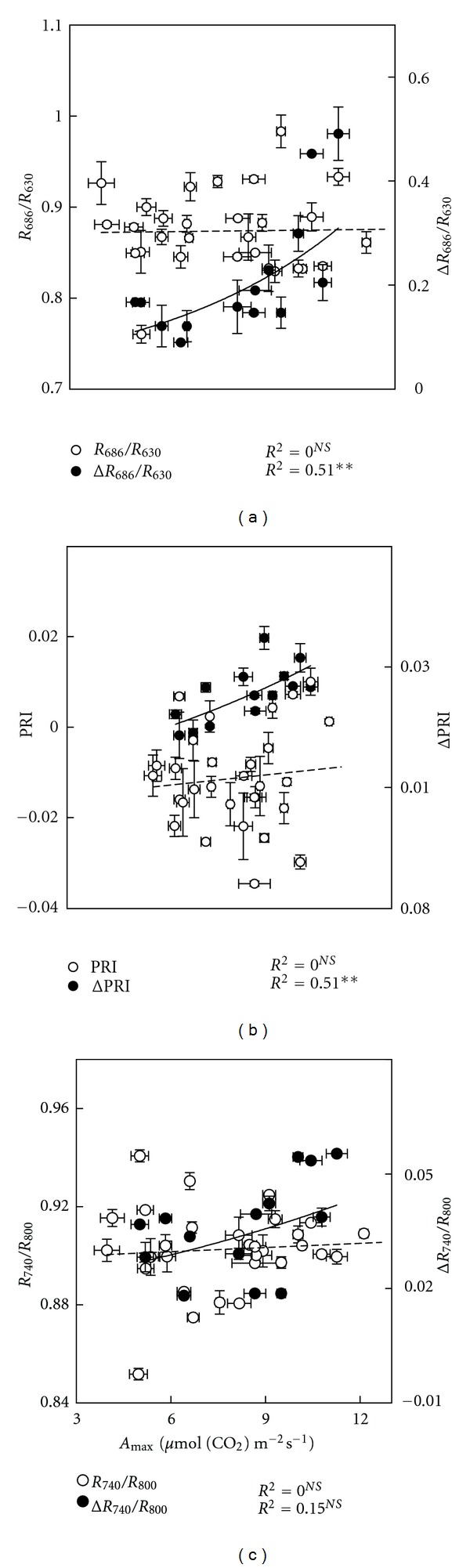
Midday relationships between light-saturated CO_2_ assimilation rate (*A*_MAX_) and selected vegetation indices ((a): *R_686_*/*R_630_*, (b): PRI Physiological Reflectance Index, (*R_532_* − *R_570_*)/(*R_532_* + *R_570_*)) and (c): *R_740_*/*R_800_* under saturating steady state light conditions (transparent dots); and the relative change of the vegetation indices (Δ) during the dynamic transition of plants form dark-to-light adapted state (dark dots). 24 leaves (of 6 different plant species) were measured and each dot represents the mean value of five measurements for *A*_MAX_ and 8–10 measurements (pixels) for the reflectance indices per one leaf. Error bars represent standard deviations. NS means nonsignificant relationship.

The relationships between *A*_MAX_ of individual leaf samples and their instantaneous VIs under steady-state saturating irradiance are shown as transparent dots in Figures 2(a)–2(c). No statistically significant relationship was found, neither between PRI nor the other reflectance ratios (*R_686_*/*R_630_*, *R_740_*/*R_800_*) and *A*_MAX_.

### 3.2. “Shade Removal” Experiment

Dark dots in Figure 2 present the relationships between *A*_MAX_ of the 24 leaf sample and the relative change of their VIs (ΔVIs) extracted from the AISA images during the dynamic transition from the dark-to-light adapted state of grassland plants. Here, significant exponential relationships (*P* < 0.05) were found between *A*_MAX_ and Δ(*R_686_*/*R_630_*) (*R* ^2^ = 0.51, *y* = 0.0484*e* ^0.1647^ *x*) (Figure 1(a)), and ΔPRI (*R* ^2^ = 0.51, *y* = 0.015*e* ^0.0621^ *x*) (Figure 1(b)). Δ(*R_740_*/*R_800_*) (Figure 2(c)) showed no significant correlation with *A*_MAX_. These results indicate that, after the inclusion of temporal dynamic of the irradiance variability in the experimental setup, the relative change in VIs (PRI and *R_686_*/*R_630_*) positively correlates with the carbon assimilation rate.

### 3.3. “Daily Course” Experiment

**Figure 3:**
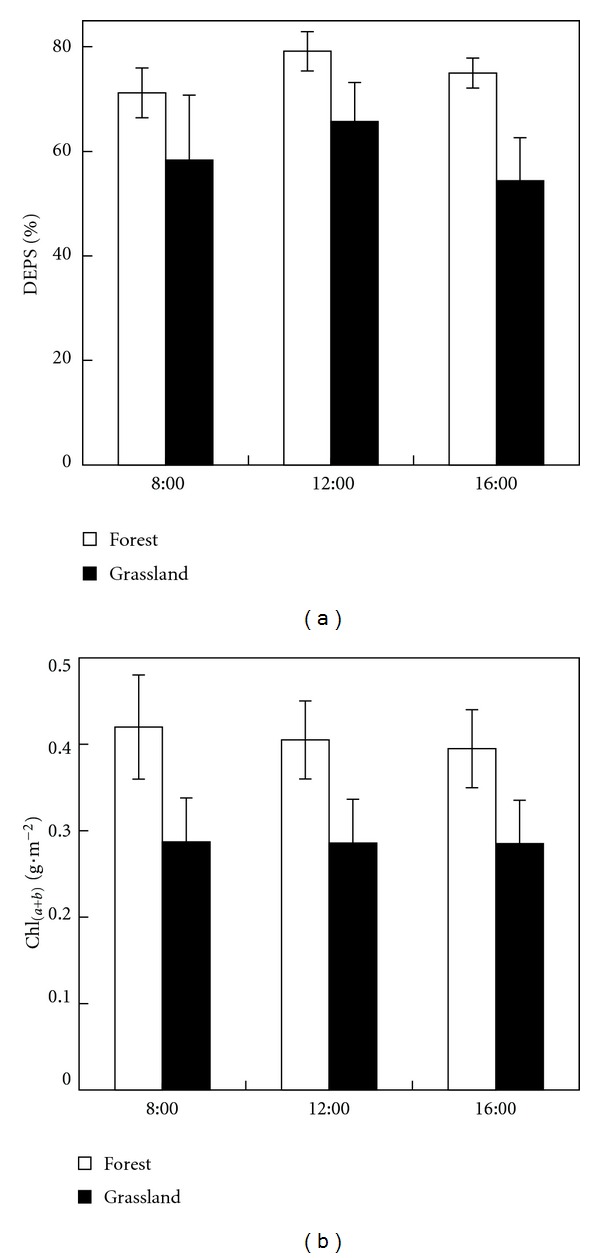
Diurnal courses of the deepoxidation state of xanthophyll cycle pigments DEPS = (Z + A)/(V + A + Z), where Z is zeaxanthin, A is antheraxanthin, and V is violaxanthin (a) and total chlorophyll pigment content (Chl_(*a*+*b*)_) (b) of grassland and forest ecosystem (black and white columns, resp.). DEPS values represent a mean value of current, 1-year-, and 2-year-old needles (*n* = 24), and Chl_(*a*+*b*)_ values represent the mean values of current needles (*n* = 6). Error bars indicate standard deviations.

Differences in the xanthophyll cycle pigments' daily variation and concentration of chlorophylls between grassland and forest stand are shown in Figure 3.  Figure 3(a) displays the daily courses of xanthophyll cycle pigments measured in the leaves of grassland plants and shoots of spruce trees. The diurnal behavior is typical for sunny days, that is, the amount of de-epoxidated xanthophyll pigments (i.e., the amount of Z and A) was highest during midday. The chlorophyll content (Chl_(*a*+*b*)_ *per unit *leaf area) was relatively during the day in both grassland as well as in forest ecosystems (Figure 3(b)). The forest canopy contained a higher relative amount of xanthophyll pigments in the de-epoxidated state (Figure 3(a)), and also a higher absolute amount of total chlorophylls (Figure 3(b)) compared to the grassland ecosystem. 

**Figure 4:**
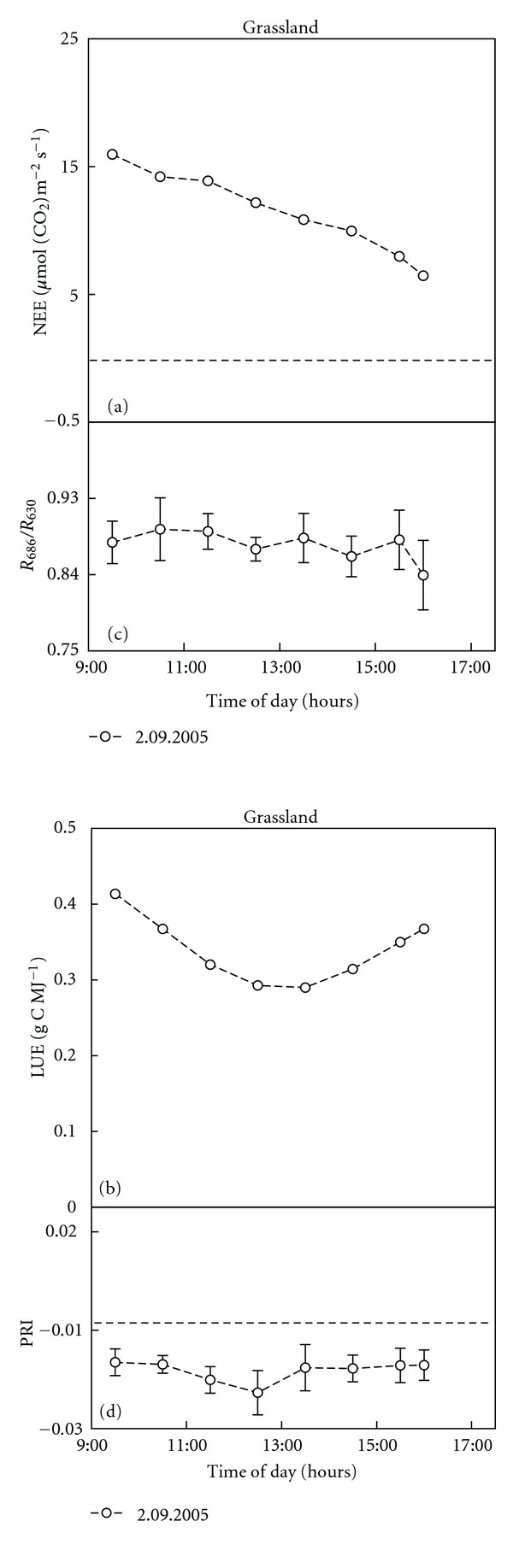
Daily courses of calculated net ecosystem exchange of CO_2_ (NEE a) and light use efficiency (LUE b) in grassland ecosystem and corresponding daily course of *R_686_*/*R_630_* (c) and Physiological Reflectance Index (PRI, (*R_532_* − *R_570_*)/(*R_532_* + *R_570_*)) (d) vegetation indexes (VIs) over experimental plot of 4 m^2^ measured via hyperspectral imaging sensor (AISA Eagle, Finland). Each data dot of eddy-covariance parameters represents an average of a half-hour measurement, and only corresponding with AISA scans are shown. VIs data dots represent an average of more than 400 000 image pixels (i.e., an average of one image scan acquired in half and one hour intervals and error bars show ± s.d.). Dashed line shows zero value of the *y*-axis.

**Figure 5:**
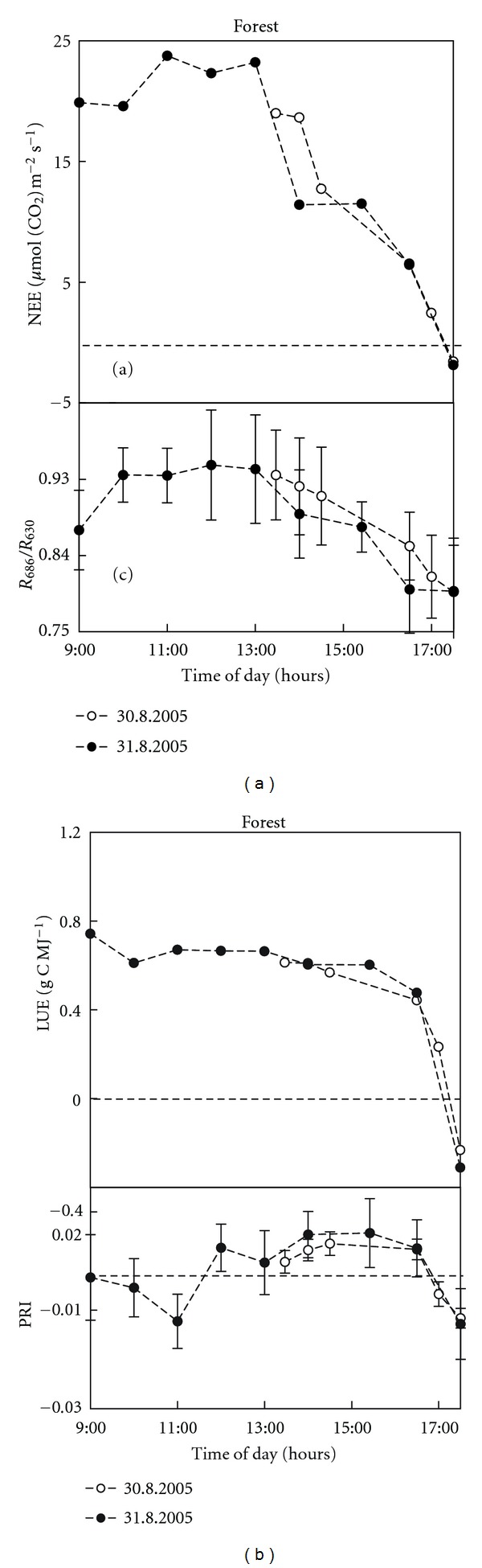
Daily courses of calculated net ecosystem exchange of CO_2_ (NEE a) and radiation use efficiency (LUE b) in forest ecosystem during two consecutive days with corresponding daily courses of *R_686_*/*R_630_* (c) and Physiological Reflectance Index (PRI, (*R_532_* − *R_570_*)/(*R_532_* + *R_570_*)) (d) vegetation indexes (VIs) measured via hyperspectral imaging sensor (AISA Eagle, Finland). Data dots of eddy-covariance parameters represent averages of half-hour measurements (a, b) and data dots of VIs (c, d) represent average of more than 400 000 image pixels (i.e., average of one image scan acquired in half and one hour intervals and error bars show ± s.d.). Dashed line shows zero value of the *y*-axis.

In this experiment, we have only presented the results on the variation of PRI and *R_686_*/*R_630_* due to their ability to follow plant dynamic changes as had been proved in the previous “shade-removal” experiment. The daily courses and also the relationships between the daily variations of PRI and *R_686_*/*R_630_*, as well as the NEE and LUE measurements of grassland and spruce forest are given in Figures 4 and 5, respectively.

In grassland, NEE gradually decreased during the day (Figure 4a), while LUE showed the typical daily course with lowest values around midday (Figure 4b). A different yet consistent pattern was observed for two consecutive days in the forest ecosystem. NEE slightly increased in the morning (till 11:00). The highest values around midday were followed by a sharp decrease in the afternoon (after 14:00) (Figure 5(a)). The daily pattern of LUE was similar for both days, showing constant values during the day and a sharp decrease after 15:00 (Figure 5(b)). Both NEE and LUE were higher in the forest compared to the grassland ecosystem. 

The daily course of the *R_686_*/*R_630_* ratio in grassland was quite stable, with the highest values at 10:30 and 11:30 (Figure 4c). In the forest *R_686_*/*R_630_* increased in the morning hours, reached the highest values during the midday hours, and then rapidly declined (Figure 5(c)). The daily patterns of PRI variations of grassland and forest measurements were similar; however, the time when PRI reached its daily minimum was shifted. The grassland PRI decreased between 10:00 to 12:30 (Figure 4(d)), reached its lowest value at the time of the highest irradiation intensity, and increased again at 13:30 to its morning values. In the forest ecosystem PRI decreased to the lowest value at 11:00 and then steeply increased, reaching maximum values from 13:30 to 15:30. In the late afternoon (15:30–17:00) it decreased again (Figure 5(d)). In grassland we were not able to record these late afternoon measurements due to the mountainous terrain and the shading of the measuring plot in the late afternoon. When comparing the absolute values, PRI for forest was higher than for grassland, which corresponds with a higher xanthophyll cycle pigment content in the de-epoxidated state of spruce needles and with a higher Chl_(*a*+*b*)_ content.

The statistically significant relationships (at *P* < 0.05) between the daily variations in *R_686_*/*R_630_* and NEE (Table 2) were found in both ecosystems. Moreover, a strong relationship was observed also between *R_686_*/*R_630_* and LUE in the forest ecosystem (Table 2). PRI showed a significant relationship (at *P* < 0.1) only with LUE in grassland (Table 2). The results of the regression analysis between the daily variations of physiological variables derived from eddy-covariance measurements and four VIs are summarized in Table 2. The ratio *R_686_*/*R_630_* showed a significant relationship with a high *R* ^2^ for both GPP and NEE in grassland, and also in the forest. Moreover, *R_686_*/*R_630_* was highly related to gLUE and LUE in the forest (Table 2), and to gLUE in grassland (Table 2). The ratio *R_740_*/*R_800_* showed a relatively high dependency on all physiological variables examined for forest but none for grassland ecosystems (Table 2).

## 4. Discussion

Our main target was to determine if VIs can indicate dynamic physiological processes related to CO_2_ assimilation rate. Under steady-state conditions, typical for RS data aquisition, we were unable to find statistically significant relationships between VIs and *A*_MAX_, indicating that a spatial scaling from the leaf up to canopy level cannot be pursued in this fashion. Grace et al. [16] pointed out that VIs related to Chl-F have the potential to provide information on the diurnal, and also on seasonal changes in photosynthesis (see also [46]). Once the temporal component with its dynamic irradiation regime had been included in our dark-to-light transition experimental setup, the variability observed in VIs increased, probably as a result of induced changes in the electron transport rate and the onset of the xanthophyll de-epoxidation processes. Consequently, also the statistically significant positive relationships between Δ(*R_686_*/*R_630_*) and ΔPRI, and *A*_MAX_ have confirmed the ability of VIs to follow the light-saturated rate of photosynthesis in the investigated species. The reflectance change at wavelengths around 690 nm was previously shown to correspond with the Chl-F emission from PSII [49, 72]. Therefore, the relative change observed in Δ(*R_686_*/*R_630_*) is comparable to the trends of the “vitality index” R_Fd_, significantly correlated with *A*_MAX_ of outdoor grown plant species [47, 73] in earlier studies. Guo and Trotter [35] published that ΔPRI for plants with contrasting photosynthetic capacities (expressed in their study as PRI at low light intensity subtracted from PRI at saturating light intensity) was negatively related to *A*_MAX_. In contrast to this, yet similar to the studies by Demmig-Adams and Adams [74], we observed a significant positive relationship, showing that a larger relative change in Z pigment content is associated with lower *A*_MAX_ in both sunlit and shaded leaves. We also observed that a larger ΔPRI was associated with a higher chlorophyll index ratio (*R_750_*/*R_710_*) [75] (see Supplementary Material available online at doi: 10.1100/2012/705872), which might explain its significant relationship with *A*_MAX_.

A number of studies [25, 29, 30, 76] investigated the possibility to use PRI derived at canopy level as a proxy for LUE in Monteith's equation [77] estimating the vegetation GPP. For instance, Rahman et al. [7] successfully used PRI derived from the Moderate Resolution Imaging Spectroradiometer (MODIS) satellite sensor for the estimation of LUE and NPP in boreal forest. However, our results showed that even if the negative effects of shade fraction and nongreen vegetation are excluded, PRI might still not be a good indicator of canopy level photosynthetic processes. Our PRI measurements were significantly related only to LUE of natural grassland and yet PRI was able to explain only 42% of LUE variability. Our result suggests that PRI relationship with LUE should be interpreted with caution, especially in ecosystems with diverse species composition, where species specific relationships might complicate the overall result.

The use of PRI at larger scales originated from leaf level studies showing that the PRI variation of leaves is driven by changes in the de-epoxidation state of xanthophyll cycle pigments in the antennae that reduce the amount of light used in leaf CO_2_ assimilation, and this way protect the leaf from excessive radiation [78, 79]. A similar trend was observed in our flux tower LUE measurements of grassland, which slightly decreased during midday. In contrast to this, the midday depression in LUE was not visible in the forest data. This might be explained by the fact that coniferous canopy contains a large proportion of shaded compared to sun exposed needles, which might be driving the LUE diurnal course. Apparently, VIs extracted from forest AISA images corresponded mainly to the sunlit shoots on top of the canopy. Our results also stressed a necessity to exclude from VI analysis the confounding effects coming from shade fraction and nongreen vegetation. All these, and other structural factors of forest canopy, can disturb a potential relationship between VIs and the eddy-covariance observations. The lack of correlation at the canopy level might, however, be also weakened by the fact that the relative changes in the pool of xanthophylls represent only a fraction of total carotenoid pigment content [80] and that light induced changes in the leaf also include some other processes, such as changes in pH of photosynthetic membrane [80] and conformational changes in chloroplasts [81]. Porcar-Castell et al. [82] provided further evidence that pool of total carotenoids is closely linked to monoterpene emission capacity, which might further complicate the interpretation of spectral response at 531 nm. Inoue and Peñuelas [83] found out that exponential relationship between LUE and PRI was strongly affected by the soil water sontent. Ollinger [84] recently summarized and highlighted structural factors, which are strongly affecting canopy level relationships between VIs and plant biophysical properties. Apart from plant physiological and structural limitations, canopy level PRI is also affected by viewing and illumination geometry [30]. Hernándes-Clemente et al. [85] found that structural sensitivity of PRI can be reduced by selecting the reference wavelength at 512 nm, instead of 570 nm. However, the experimental results of our study found a significant relationship between canopy VIs and CO_2_ assimilation-related plant physiological variables when considering their daily cycle variations. Obtained *R* ^2^ coefficients between PRI and LUE are lower than the ones reported in the recent meta-analysis [17]. This might be caused by a narrower dynamic range of PRI and LUE measured during our experiment.

The narrow-band reflectance ratios of Chl-F wavelengths (*R_686_*/*R_630_*, *R_740_*/*R_800_*) performed better than PRI, and stronger relations were observed for forest than for grassland canopy. This might be partially explained by lower forest water stress, which increased the leaf stomatal conductance resulting in a stronger Chl-F emission. Although most of the studies investigated fluorescence VIs in relation to various Chl-F-related parameters [40, 49, 50], several studies reported a positive relationship of photosynthesis-related variables with reflectance ratios related to Chl-F. The laboratory experiment of Dobrowski et al. [37] resulted in a positive relationship between the reflectance ratio *R_690_*/*R_600_* and CO_2_ assimilation (*R* ^2^ = 0.36). Furthermore, the reflectance ratio *R_695_*/*R_805_* measured in a 5-year-old canopy of pine seedlings was significantly related to *A*_MAX_ [86] during the diurnal course. In general, our results showed higher *R* ^2^ values of the reflectance ratios to LUE than to gLUE, which can be explained by the respiration component included in the “gross” measurements. The reflectance ratio *R_686_*/*R_630_*, tested in this study, was found to be a statistically significant proxy of GPP, NEE, and LUE in grassland and also of gLUE in forest ecosystems. Thus, we can hypothesize that *R_686_*/*R_630_* might be considered a potential RS indicator of the ecosystem vegetation productivity variables.

## 5. Conclusions

The first experimental results conducted at the leaf level revealed no statistically significant relation of “process-related” narrow-band VIs (PRI; *R_686_*/*R_630_*; *R_740_*/*R_800_*) to the maximum CO_2_ assimilation rate measured instantaneously under the steady-state irradiation conditions. Significant correlations were only found once the relationships between VIs and physiological variables were investigated at a temporal scale (minutes and hours), taking into account the changes in irradiance intensity. When taking into account the 1.5-minute duration of the dark-to-light transition, Δ(*R_686_*/*R_630_*) and ΔPRI of natural grassland, we were able to establish significant regressions between VIs and the carbon assimilation measured under saturating light conditions. Finally, in the daily course of several hours, the simple reflectance ratio *R_686_*/*R_630_*, of the VIs investigated, showed the highest *R* ^2^ value to the measured eddy-covariance ecosystem variables with better statistical relations found in the case of spruce forest than in grassland canopy. Considering that we observed a strong statistical relation to GPP and NEE, we conclude that the *R_686_*/*R_630_* ratio may potentially act as a suitable RS indicator of vegetation productivity at the scale of the whole canopy. In general, the results of this study provide an insight relevant to the further development of leaf-canopy upscaling remote sensing approaches as well as basis for more accurate estimation of carbon assimilation and the productivity in natural canopies. However, considering spatial and temporal limitations of the presented study, many problems are yet to be resolved before a remote sensing application at larger spatial scales.

## Supplementary Material

Supplementary Figure: Relationship between the Photochemical Reflectance Index (PRI, (*R*_532_ – *R*_570_)/(*R*_532_ + *R*_570_)) and chlorophyll index (*R*_750_/*R*_710_) (a) and between PRI (●) and ΔPRI (*◍*) and *R*_750_/*R*_710_ (b). Asterisks denotes for statistical significance (∗∗*P* < 0.001), error bars show ± SD (*n* = 8–10).

## Figures and Tables

**Table 1:** Vegetation indices (VIs) used in this study. *R*_*λ*_ is reflectance at a wavelength *λ* in nm, [L.UE: light use efficiency; F_S_: *steady-state* chlorophyll fluorescence; Chl*_a+b_* total chlorophyll content].

Vegetation index	Designed as indicator of	Reference
PRI = $\frac{\left(R_{532.2}-R_{570.8}\right)}{\left(R_{532.2}+R_{570.8}\right)}$	LUE, Zeaxanthin content,	[23]
*R_686.7_*/*R_630.0_*	F*_S_*, early stress	[50]
*R_740.0_*/*R_800.0_*	F*_S_*, early stress	[37]

**Table 2:** Summary of coefficients of determination (*R^2^*) found for the best-fit between vegetation indices (VIs) and physiological parameters derived from eddy-covariance measurements in grassland and forest ecosystems. Gross primary production (GPP [*μ*mol (CO_2_) m^−2^ s^−1^] = NEE + R); net ecosystem exchange of CO_2_ (NEE [*μ*mol (CO_2_) m^−2^ s^−1^]); gross light use efficiency (gLUE = GPP/PPFD [g C MJ^−1^]); radiation use efficiency (LUE = NEE/PPFD [g C MJ^−1^]). Values of eddy-covariance derived parameters are half-hour averages and values of VIs are averages of more than 400 000 pixels (i.e., average of one image scan acquired in half to one hour intervals), *R* is reflectance at subscripted wavelength and PRI is calculated as (*R_532_* − *R_570_*)/(*R_532_* + *R_570_*). *Denotes *P* < 0.1 and ** for *P* < 0.05, as determined by ANOVA analysis.

Grassland (*n* = 8)					Forest (*n* = 15)			
VIs	GPP	NEE	gLUE	LUE	GPP	NEE	gLUE	LUE
PRI	0.20	0.00	0.20	0.42*	0.08	0.07	0.08	0.38
*R_686_*/*R_630_*	0.49*	0.42*	0.49*	0.00	0.88**	0.85**	0.88**	0.58**
*R_740_*/*R_800_*	0.02	0.07	0.02	0.05	0.71**	0.69**	0.83**	0.52*
