# Supplementary figures and images for: Relation of Chlorophyll Fluorescence Sensitive Reflectance Ratios to Carbon Flux Measurements of Montanne Grassland and Norway Spruce Forest Ecosystems in the Temperate Zone
Source: ScientificWorldJournal. 2012 Jun 4;2012:705872. doi: 10.1100/2012/705872 (PMC3373153; doi:10.1100/2012/705872)

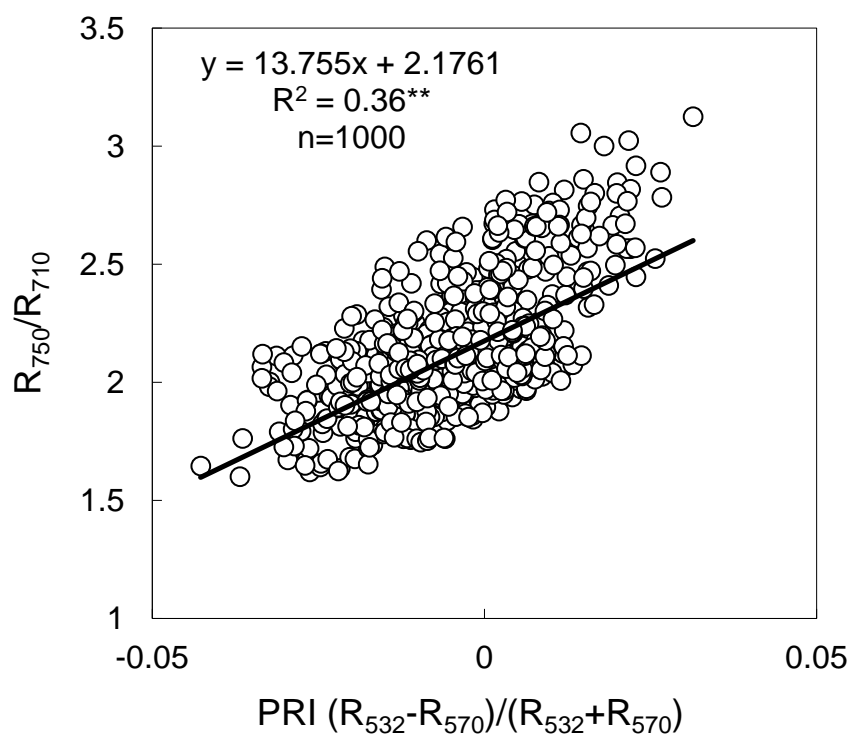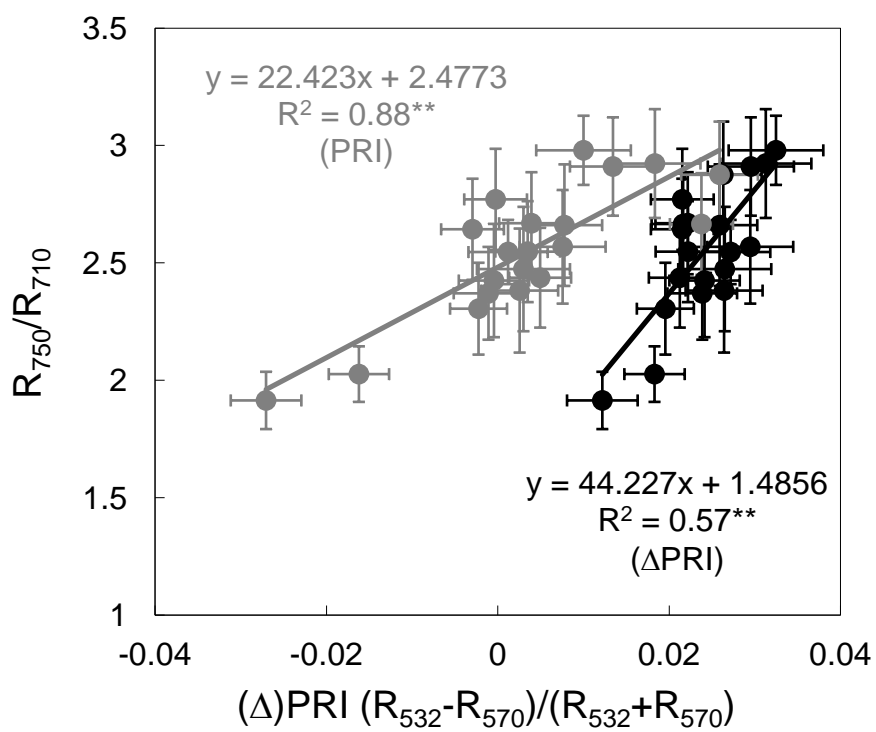

Supplement: Supplementary file 1 — Supplementary Figure: Relationship between the Photochemical Reflectance Index (PRI, (R 532 – R 570)/(R 532 + R 570)) and chlorophyll index (R 750/R 710) (a) and between PRI (●) and ΔPRI (◍) and R 750/R 710 (b). Asterisks denotes for statistical significance (∗∗P < 0.001), error bars show ± SD (n = 8–10). [file 705872.f1.pdf]
